# ﻿*Cryptotermespugnus* (Blattodea, Isoptera, Kalotermitidae), a new drywood termite species from the Brazilian Caatinga dry forest and key to South American *Cryptotermes* Banks, 1909

**DOI:** 10.3897/zookeys.1182.108243

**Published:** 2023-10-11

**Authors:** Rudolf H. Scheffrahn, Alexandre Vasconcellos

**Affiliations:** 1 Fort Lauderdale Research and Education Center, University of Florida, 3205 College Avenue Davie, Florida 33314, USA University of Florida Davie United States of America; 2 Laboratório de Termitologia, Departamento de Sistemática e Ecologia, Centro de Ciências Exatas e da Natureza, Universidade Federal da Paraíba, Paraíba, Brazil Universidade Federal da Paraíba João Pessoa Brazil

**Keywords:** Arolium, Bahia, imago, Paraíba, soldier, South America, venation

## Abstract

A new termite species, *Cryptotermespugnus***sp. nov.**, is described from northeastern Brazil. The winged imago of *C.pugnus* is distinguished from most congeners by the lack of arolia and the multiple branches connecting the median vein to the radial sector. The soldier is unique among South American *Cryptotermes* by its cuboidal head capsule and very rugose postclypeus. The new species constitutes the fourteenth *Cryptotermes* species on the continent for which we provide a key to soldiers.

## ﻿Introduction

The cosmopolitan termite genus *Cryptotermes* Banks, 1906 is most diverse in the Neotropics with 32 of the 72 species described worldwide (Constantino 2020). Three of the Neotropical species are exotic pests, including *C.brevis* (Walker, 1853) (only the populations outside its endemic region of coastal Chile and Peru), *C.dudleyi* Banks, 1918, and *C.havilandi* (Sjostedt, 1900) with a previous fourth, *C.domesticus* Haviland, 1898, now deemed absent from the New World (Scheffrahn et al. 2009; Scheffrahn 2021). Until now, mainland South America (and Trinidad and Tobago) was habitat to 11 endemic *Cryptotermes* species: *C.aequacornis* Scheffrahn & Křeček, 1999; *C.brevis*; *C.camelus* Scheffrahn, 2021; *C.chacoensis* Roisin, 2003; *C.colombianus*Casalla et al., 2016; *C.contognathus* Constantino, 2000; *C.cubicoceps* (Emerson, 1925); *C.cylindroceps* Scheffrahn & Křeček, 1999; *C.mangoldi* Scheffrahn & Křeček, 1999; *C.rhicnocephalus* Bacchus, 1987; and *C.verruculosus* (Emerson, 1925). *Cryptotermesmangoldi* and *C.cylindroceps* were originally described from the West Indies until Casalla et al. (2016) reported their mainland distribution. Here, we describe a new endemic mainland species, *C.pugnus* sp. nov., from northeastern Brazil and provide a key to the described *Cryptotermes* from South America.

## ﻿Material and methods

Photomicrographs were taken as multilayer montages using a Leica M205C stereomicroscope controlled by Leica Application Suite v. 3 software. Preserved specimens were taken from 85% ethanol and suspended in a pool of Purell Hand Sanitizer to position the specimens on a transparent Petri dish background. Comparisons with other South American *Cryptotermes* species were made from specimens in the University of Florida Termite Collection (Scheffrahn 2019).

## ﻿Taxonomy

### ﻿ Cryptotermes pugnus

Taxon classificationAnimaliaBlattodeaKalotermitidae

Scheffrahn & Vasconcellos sp. nov.

A37D1611-D683-52A6-881E-9E18BB014DB4

https://zoobank.org/B7EB068D-34FD-4B36-A5B9-C37A230F05FD

#### Comparison.

**Figure 1.**
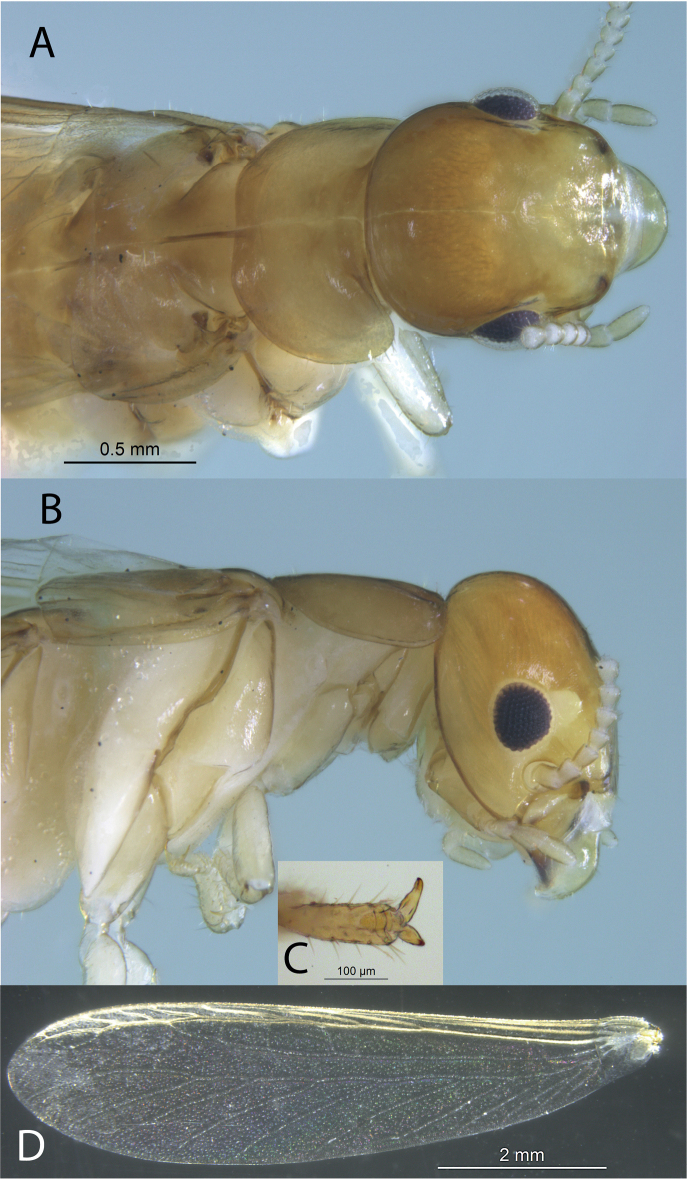
Imago of *Cryptotermespugnus* sp. nov. (SA470) **A** dorsal view of head and pronotum **B** lateral view of head and pronotum **C** distal tarsomere of foreleg **D** left forewing.

The imago of *C.pugnus* groups with *C.brevis*, *C.chacoensis* Roisin, 2003, *C.kirbyi* Moszkowski, 1955, and *C.darwini* (Light, 1935) in having the arolium absent between the tarsal claws (Fig. 1C). The forewing venation of the *C.pugnus* imago is atypical for most of the genus in having several branches splitting from the media and intersecting the radial sector (Fig. 1D). This character is only known from *C.brevis*, *C.darwini* (see Light 1935), and *C.kirbyi* (see Bacchus 1987). The latter two species may be found in future studies to by synonyms of *C.brevis*. Roisin (2003) did not describe the venation of *C.chacoensis*.

**Figure 2.**
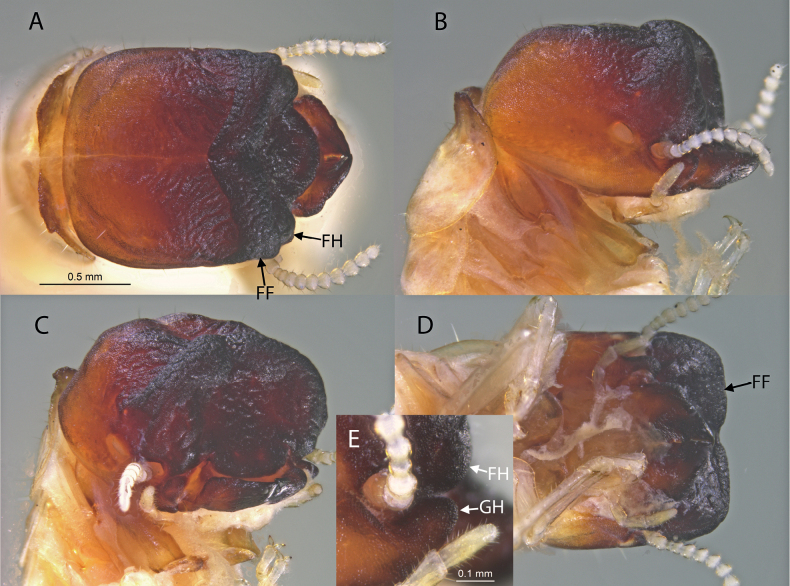
Soldier of *Cryptotermespugnus* sp. nov. (SA470) **A** dorsal view of head and pronotum **B** lateral view of head and pronotum **C** oblique view of head and pronotum **D** ventral view of head and pronotum **E** lateral view of cephalic horns. FF = frontal flange, FH = frontal horn, and GH = genal horn.

**Figure 3.**
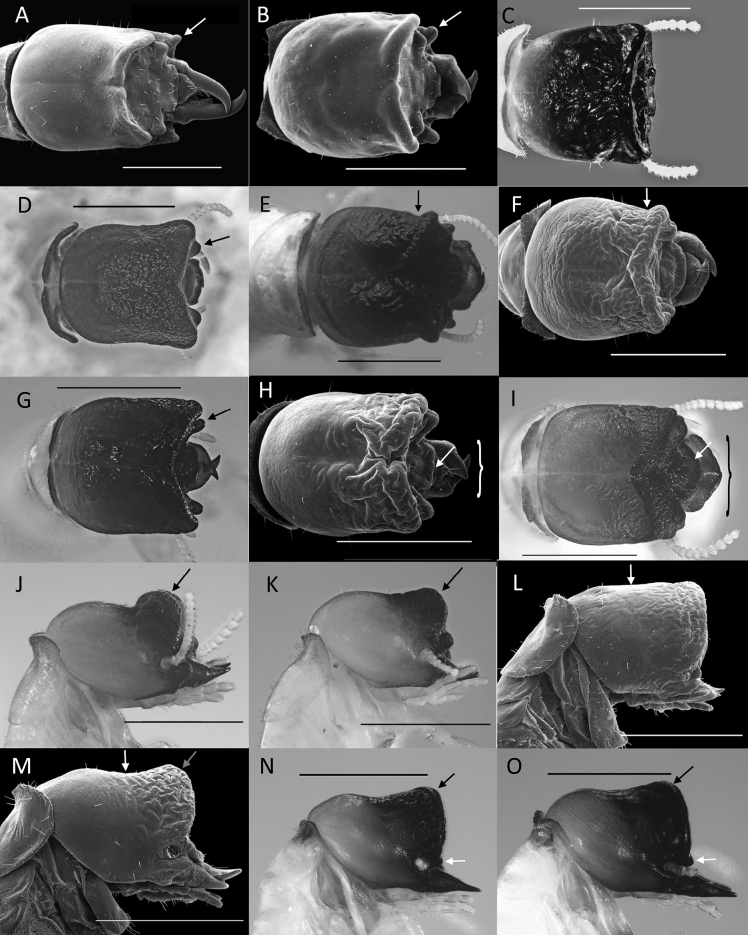
South American *Cryptotermes* soldier head capsules **A***C.dudleyi* (arrow: genal horn) **B***C.havilandi* (arrow: genal horn) **C***C.colombianus***D***C.contognathus* (arrow: frontal horn) **E***C.chacoensis* (arrow: constriction) **F***C.brevis* (arrow: constriction) **G***C.cubicoceps* (arrow: frontal horn) **H***C.aequacornis* (arrow: postclypeus; bracket: outer span of mandibles) **I***C.pugnus* sp. nov. (arrow: postclypeus; bracket: outer span of mandibles) **J***C.camelus* (arrow: frontal flange) **K***C.verruculosus* (arrow: frontal flange) **L***C.cylindroceps* (arrow: lateral margin of vertex) **M***C.rhicnocephalus* (white arrow: vertex concave, grey arrow: frontal flange without elevated rim) **N***C.fatulus* (black arrow: frontal flange with elevated rim; white arrow: frontal horn) **O***C.mangoldi* (black arrow: frontal flange with elevated rim; white arrow: frontal horn with elevated rim). **A, B, F, H, L, M** modified from Scheffrahn and Křeček (1999); C modified from Casalla et al. (2016). Scale bars: 1 mm.

Among mainland South American *Cryptotermes* soldiers, *C.pugnus* is unique in having, in dorsal view, a cuboidal head capsule and a very rugose, rounded and projecting postclypeus (Fig. 2). The postclypeus of *C.brevis* and *C.chacoensis* soldiers are closest to *C.pugnus*, but the head capsules of the former two are constricted (Fig. 3). Along with *C.pugnus*, only *C.aequacornis*, *C.cylindroceps*, and *C.rhicnocephalus* have both frontal and genal horns projecting the same length anteriorly (Fig. 3H, L, M).

#### Description.

***Imago*** (Fig. 1A–D). Head capsule and pronotum pale yellow brown. Compound eye obtusely triangular; ocellus light yellow, about half diameter of eye, roundly ellipsoid, and touching eye margin. Vertex with a few short setae. Pronotum wider than head capsule; anterior margin shallowly concave. Pronotum lateral margins with about one dozen setae each. Antennae with 15 articles, basal article relative lengths 2 = 3 > 4 = 5. Forewing with subcosta joining costal margin at about 1/8 of wing length from suture. Wing membrane pale; veins a shade darker. Costa, subcostal, radius, and radial sector sclerotized; unsclerotized media with several branches intersecting radial sector; media terminating at radial sector about 3/4 wing length, then appearing as a separate branch near tip of wing. Arolium absent. Measurements (mm, mean, *n* = 3). Head maximum width with eyes 0.96; head maximum width without eyes 0.88; pronotum maximum width 0.94; eye maximum diameter 0.23; ocellus maximum diameter 0.12; total body length 5.3; right forewing length from scale 6.90; body length with wings 8.74.

***Soldier*** (Fig. 2A–E). Head capsule, in dorsal view, strongly rugose; dark castaneous brown from postclypeus grading to orange-brown at occiput. Head capsule widest at posterior third, narrowest at frontal flange. Frontal flange (ridge) V-shaped with deep median cleft. Posterior margin of head capsule truncate, posterolateral corners forming right angles, lateral margins nearly parallel combining to form cuboidal appearance. In lateral view, frontal flange elevated, vertex unevenly concave; frontal horns visible as blunt knobs. Genal horns evenly rounded, slightly posterior to frontal horns (Fig. 2E). Pronotum angled sharply from vertex, narrower than head; anterior margin dark and ruffle; incised in middle with rounded anterior lobes. Eye spots large, narrowly elliptical. In oblique view (Fig. 2C) frons concave. Postclypeus evenly convex, strongly rugose, projecting well beyond frontal flange (Fig. 2A). Antennae with 8 or 9 articles, third fused or divided; or with 10 or 11 articles, third fused or divided. Mandibles wide and short for the genus; rugose, rounded basal hump at half-length when seen from below, outer margin of blade angles about 50°. Measurements (mm, mean, *n* = 2). Head length to tip of mandibles 1.57; head length to tip genal horns 1.20, frontal flange width 1.11; frontal horns, outside span 0.90; genal horns, outer span 0.95; head width, maximum 1.20; head width, minimum (behind frontal flange) 1.10; head height, excluding postmentum 0.88; pronotum, maximum length 0.95; pronotum, maximum width 1.12; left mandible length, tip to ventral condyle 0.53.

#### Type materials.

***Holotype***: Brazil • Soldier; Paraíba, São José dos Cordeiros; -7.39056, -36.80833; 526 m a.s.l.; 17 Aug. 2000; A. Vasconcellos leg.; two soldiers (one labelled holotype, Fig. 2), three imagos, and three pseudergates; University of Florida Termite Collection (UFTC) no. SA470, subsample from Federal University of Paraíba Termite Collection (FUPTC) no. 2052. ***Paratypes***: Brazil • Bahia, Curaçá; −9.123, −39.691; 366 m a.s.l.; 4 May 2011; A. Vasconcellos leg.; one soldier and pseudergates; FUPTC no. 4345.

#### Etymology.

Named after the pug dog. The oblique view of the soldier (Fig. 2C) resembles this short-nosed breed.

##### ﻿Key to South American *Cryptotermes* soldiers

**Table:** 

1	In dorsal (or ventral) view, genal horns form anterolateral knobs of head capsule; vertex smooth (introduced species) (Fig. 3A, B)	**2**
—	In dorsal view, genal horns eclipsed by frontal horn or frontal flange (e.g. Fig. 3H)	**3**
2	Mandibles project more than one third length of head capsule (Fig. 3A)	***C.dudleyi***
—	Mandibles project about one fourth length of head capsule (Fig. 3B)	***C.havilandi***
3	Mandibles barely project beyond frons or frontal horns (Fig. 3C, D)	**4**
—	Mandibles clearly project beyond frons or frontal horns (e.g. Fig. 3H)	**5**
4	Frontal horns not visible (Fig. 3C)	***C.colombianus***
—	Frontal horns visible (Fig. 3D)	***C.contognathus***
5	Vertex excavated; with deeply folding rugosity (e.g. Fig. 3H)	**6**
—	Vertex not excavated; rugosity more shallow (e.g. Fig. 3M)	**10**
6	Head constricted behind frontal flange (Fig. 3E, F)	**7**
—	Head not constricted behind frontal flange (Fig. 3G–I)	**8**
7	Genal horns visible from above, mandibles with lateral humps; Gran Chaco region (Fig. 3E)	C.chacoensis ^*^
—	Genal horns not visible from above, mandibles without lateral humps; widespread (Fig. 3F)	C.brevis ^*^
8	Frontal horns barely extend beyond anterolateral margin of frontal flange (Fig. 3G)	***C.cubicoceps***
—	Frontal horns extend well beyond anterolateral margin of frontal flange (Fig. 3H, I)	**9**
9	Anterior margin of postclypeus linear; outer span of mandibles <1/2 width of head (Fig. 3H)	***C.aequacornis***
—	Anterior margin of postclypeus rounded; outer span of mandibles >1/2 width of head (Fig. 3I)	***C.pugnus* sp. nov.** ^*^
10	In lateral view, frontal flange emerges above vertex as a rounded mound (Fig. 3J, K)	**11**
—	In lateral view, frontal flange forms angular intersection with vertex (Fig. 3L–N)	**12**
11	Frontal flange semicircular; humid Chaco (Fig. 3J)	***C.camelus***
—	Frontal flag quadrant (Fig. 3K)	***C.verruculosus***
12	Lateral margin of vertex linear in lateral view (Fig. 3L)	***C.cylindroceps***
—	Lateral margin of vertex concave (Fig. 3M–O)	**13**
13	Flange without elevated rim (Fig. 3M)	***C.rhicnocephalus***
—	Flange with elevated rim (Fig. 3N, O)	**14**
14	Frontal horn not projecting beyond frontal flange (Fig. 3N)	***C.fatulus***
–	Frontal horn projects beyond frontal flange (Fig. 3O)	***C.mangoldi***

## ﻿Discussion

*Cryptotermespugnus* is the second species of the genus described from Brazil and the first from the Caatinga dry forest, with records for two ecoregions, which have different geomorphological history and climatic parameters, “Planalto da Borborema” (São José dos Cordeiros, Paraíba State) and “Depressão Sertaneja Meridional” (Curaçá, Bahia State) (Silva et al. 2018). There is also a record of *C.havilandi* from the Caatinga dry forest (Vasconcellos unpublished data), an exotic species which probably originated in tropical West Africa (Scheffrahn et al. 2003). There are no records of *C.pugnus* infestations in buildings, either in urban or agricultural environments. Other kalotermitids reported from the Caatinga include two undescribed species of *Glyptotermes* Froggatt, 1897, Rugitermescf.niger Oliveira, 1979, an undescribed species of *Rugitermes* Holmgren, 1911 (Bandeira et al. 2003), and *Tauritermesbandeirai* Scheffrahn & Vasconcellos, 2022 (Scheffrahn and Vasconcellos 2020).

Small colonies of *C.pugnus* were found on adult individuals of *Cenostigmanordestinum* E. Gagnon & G.P. Lewis, an endemic tree of the Caatinga dry forest, which presents hard, highly dense (>0.84 g/cm^3^) wood and individuals that can exceed 10 m in height (Silva et al. 2009). Due to the hardness of the wood, access to *C.pugnus* colonies is difficult, requiring the use of an ax and/or chainsaw. Possibly because of this, its colonies are rarely found. At the type locality, there are records of *C.pugnus* alate flights from late December to early February (Lucena et al. 2022).

## Supplementary Material

XML Treatment for Cryptotermes pugnus

## Footnotes

Imago without arolia (C. camelus imago unknown).

## ﻿Additional information

### Author ORCIDs

Alexandre Vasconcellos https://orcid.org/0000-0001-7211-7097

### Data availability

All of the data that support the findings of this study are available in the main text.
